# Application of Standardized Proportional Mortality Ratio to the Assessment of Health Risk in Relatively Healthy Populations: Using a Study of Cancer Risk in Telecommunication Workers with Excess Exposure to Acid Mists as an Example

**DOI:** 10.3390/ijerph18189870

**Published:** 2021-09-19

**Authors:** Ying-Fong Ker, Perng-Jy Tsai, How-Ran Guo

**Affiliations:** 1 Department of Environmental and Occupational Health, College of Medicine, National Cheng Kung University, Tainan 704, Taiwan; brgker@gmail.com (Y.-F.K.); pjtsai@mail.ncku.edu.tw (P.-J.T.); 2 Los Angeles County Department of Public Health, Alhambra, CA 91803, USA; 3 Department of Occupational and Environmental Medicine, National Cheng Kung University Hospital, Tainan 704, Taiwan

**Keywords:** acid mist, healthy worker effect, neoplasm, occupational exposure, standardized mortality ratio, standardized proportional mortality ratio, stomach neoplasms, sulfuric acid

## Abstract

When a study population is relatively healthy, such as an occupational population, epidemiological studies are likely to underestimate risk. We used a case study on the cancer risk of workers with exposure to acid mists, a well-documented carcinogen, to demonstrate that using proportional mortality ratios (PMRs) is more appropriate than mortality ratios in assessing risk in terms of mortality. The study included 10,229 employees of a telecommunication company who worked in buildings with battery rooms. In these buildings, the battery rooms had the highest levels of sulfuric acid in the air (geometric mean = 10.7 μg/m^3^). With the general population in Taiwan as a reference, a decreased standardized mortality ratio (0.42, *p* < 0.01) from all causes combined, between 1 January 1985 and 31 December 1996, was observed, indicating a healthy worker effect. When we reanalyzed the data using standardized PMR, elevated risks were observed for all cancers combined (1.46, *p* = 0.01) and cancers of the digestive organs and peritoneum (1.61, *p* = 0.02), especially stomach cancer (2.94, *p* = 0.01). The results showed that PMR can detect increases in mortality when a study population is generally healthier than the comparison population and call for further studies on the possible carcinogenic effects of low-level acid mist exposures on the stomach.

## 1. Introduction

In epidemiological studies, when the study population is relatively healthier than the comparison population, risk is likely to be underestimated. A typical scenario is that an occupational population is likely to be healthier than the general population because it comprises individuals necessarily healthy enough to be employable. Consequently, when a study evaluates effects of a health hazard using mortality as the indicator of outcome, if an occupational population with exposure to that health hazard is compared to the general population, the risk associated with the health hazard is likely to be underestimated. This phenomenon is often called the “healthy worker effect” [1]. 

Standardized mortality ratio (SMR) is a frequently used measure of the relative risk of mortality, expressing the mortality experience of the study population relative to that of a comparison (standard) population. With the general population as the standard, the SMR for a study population that is relatively healthier (such as an occupational population) will underestimate the mortality experience of the study population, because the baseline mortality risk is initially lower than that of the standard population. We hereby use a case study on the cancer risk of workers with exposure to acid mists, a well-documented carcinogen, to demonstrate that using proportional mortality ratios (PMRs) is more appropriate than SMR in such cases.

Strong inorganic acids, including sulfuric acid, hydrogen acid, and nitric acid, are commonly used in industry. Long term exposure to high-levels of strong inorganic acid mists is a well-documented risk factor for developing cancer, especially in the upper respiratory tract, such as lung and laryngeal cancers among steel workers, while the International Agency for Research on Cancer (IARC) classified it as a Class I carcinogen (carcinogenic to humans) [2]. An increased risk of cancers of the upper aerodigestive tract was also observed in workers exposed to acid mists [3]. Most previous studies on the carcinogenic effect of acid mists were conducted in workplaces with relatively high levels of exposure, mainly through the application of strong inorganic acids in the manufacturing process. For example, the average exposure levels in the study by Beaumont et al. [4] and the study by Steenland et al. [5] were both around 200 μg/m^3^. While this level was below the 1000 μg/m^3^ standard proposed by the Occupational Safety and Health Administration of the United States, excess risks of cancer were still observed. To assess the risk associated with even lower exposure levels, a study was conducted on the workers of a large telecommunication company in Taiwan, who had worked in buildings with battery rooms [6]. The batteries were used as back-up power supplies and contained strong acids, mainly sulfuric acid, and workers in such buildings were exposed to acid mists transported through the ventilation system. 

## 2. Materials and Methods

### 2.1. Study Population and Exposure Assessment

The study population consisted of employees of a large telecommunication company in Taiwan, who had worked in buildings with battery rooms. In Taiwan, before 1996, telecommunication was a monopoly business run by the government, and the company offered very good benefits, including the salary and health insurance, in comparison with other companies in general. Therefore, its employees constituted a very healthy population compared to the general population and thus provide a good example for demonstrating the healthy worker effect.

The batteries are used as back-up power supplies in case of power shortage, and they are generally placed in battery rooms, which no workers occupied on a regular basis. These large batteries contain strong acids, mainly sulfuric acid. During the charging process, both hydrogen and oxygen bubbles can be generated, and the bursting of gas bubbles on top of the acid solution may lead to the release of aerosols containing acids. Whereas it is difficult to estimate the particle sizes of these aerosols, a laboratory study on aerosols generated from chromic acid solutions with various gas (bubble) flow rates indicated that the distribution of particle sizes was bimodal [7]. The mass median aerodynamic diameter observed in the fine mode and the coarse mode was <4 µm and 10~15 µm, respectively. As the gas flow rate in the battery cell should be much less than that observed in the above laboratory setting, the aerosols generated during the charging process of batteries would be even smaller in size. Therefore, the acid aerosols emitted from batteries can be inhaled by humans. Although only small amounts of acid mists are given off from the batteries, they can be transported throughout the whole building through the ventilation system. Therefore, although almost all members of the study population were office workers, they were exposed to certain levels of acid mists in the work environment. 

Sampling was conducted in 23 typical buildings, using a sampling train consisting of one inhalable aerosol sampler and followed by one sorbent tube, for collecting acidic aerosols and vapors, respectively. The obtained exposure levels were generally lower than those studied in other occupational populations. The highest environmental levels of sulfuric acid were measured in the battery rooms (3.05 to 32.6 μg/m^3^, geometric mean = 10.7 μg/m^3^), but they were all within the regulatory standard of 1000 μg/m^3^. Although we did not measure acid mist levels in all the buildings covered in this study, because all the building belonged to the same company, who had built most of them, the levels were generally within this range. In addition to acid mists, the levels of electromagnetic field and heavy metals in the buildings with battery rooms were assessed. The mean air level was 0.844 μg/m^3^ for chromium, 0.002 μg/m^3^ for cadmium, 1.21 μg/m^3^ for lead, and 0.804 μg/m^3^ for nickel, and were all far below the regulatory standards in Taiwan (500 μg/m^3^ for chromium, 50 μg/m^3^ for cadmium, 100 μg/m^3^ for lead, and 100 μg/m^3^ for nickel). 

### 2.2. Follow-Up of the Study Cohort

Data on the follow-up of the study population were adopted from a previous project [6]. From personnel records, the project identified the employees who had worked in buildings with battery rooms for one year or more from 1 January 1985 to 31 December 1996 as the study cohort. For each cohort member, information on sex, date of birth, working unit, date of arriving at the unit, and date of leaving the unit was obtained. 

The project used the National Mortality Registry operated by the Ministry of Health to follow up the vital status of all cohort members from 1 January 1985 to 31 December 1996. The computerized records of the registry include information on age, sex, date of birth, occupation, date of death, cause of death (disease coded according to the ninth edition of the International Classification of Disease; ICD-9), and township of resident of each case. In Taiwan, the prompt reporting of deaths is mandated by law, which ensures the completeness of the registry. 

### 2.3. Data Analysis

The SMR of a given cause of death for the study cohort was calculated using the observed number of deaths as the numerator and the expected number of deaths as the denominator [8]. The person-years of follow-up of all cohort members were summarized by sex, age, and calendar year, and the expected number of cases were calculated based on the cancer risk observed in the whole Taiwan population (as the standard population), because the company had buildings with battery rooms all over Taiwan. All cohort members were followed till death or 31 December 1996, even after they left the company. Deaths with ICD-9 codes 140 to 208 were classified as cancer deaths. To take into account the latency period of cancer, a further analysis was conducted on person-years of follow-up after the first five years of employment. The length of this period was chosen based on previous studies [3,9]. 

In addition to SMR, we used standardized PMR (SPMR) to assess the cancer risk. While the SPMR is also obtained by dividing the observed number of cases by the expected number of cases, the expected number of cases is calculated on the basis of the proportion of death due to a specific cause among all deaths in the standard population [8]. Again, the population of the whole Taiwan area was used as the standard population. 

All statistical analyses were performed using statistical analysis software (SAS, Version 6.12, SAS Institute, Cary, NC, USA), and the statistical significance of SMRs and SPMRs was evaluated by exact tests based on the Poisson distribution at a significant level of 0.05. 

## 3. Results

A total of 10,229 workers that fit our cohort definition were identified, including 9551 men (93.4%) and 678 women (6.6%). The total person-years at follow-up were 106,725, and 155 deaths were observed between 1 January 1985 and 31 December 1996. Among those, 52 died of cancers, including 31 of cancers of the “digestive organs and peritoneum” (Table 1). There were seven deaths due to cancers of the “respiratory and intrathoracic organs,” and all of them were lung cancers. Likewise, all the five deaths due to cancers of “lip, oral cavity, and pharynx” were nasopharyngeal cancers. No cases of laryngeal cancer were observed. 

The SMR for all causes combined was 0.42, and that for all cancers combined was 0.64; both were statistically significant (both with *p* < 0.01, Table 1). Among cancers with a specific three-digit ICD-9 code and with observed deaths, only cancers of the “liver and intrahepatic bile ducts” had a statistically significant SMR (0.52, *p* = 0.01), indicating a lower than expected (decreased) risk. For the other three leading causes of death in Taiwan, the SMR was 0.55 for cerebrovascular diseases (*p* = 0.01), 0.28 for accidents (*p* < 0.01), and 0.65 for cardiovascular diseases (*p* = 0.08). 

When person-years within the first five years of employment were excluded, a total of 122 deaths, including 40 due to cancers, were observed among the 70,266 person-years at risk. As a result, the SMR generally increased (Table 1). The SMR for all causes of death combined was 0.46 (*p* < 0.01), and that for all cancers combined was 0.69 (*p* = 0.01). However, changes in the SMR of specific cancers varied. For example, the SMR for cancers of “lip, oral cavity, and pharynx” and “respiratory and intrathoracic organs” increased, but that for cancers of the “digestive organs and peritoneum” decreased. Even among cancers of the “digestive organs and peritoneum,” whereas the SMR for cancers of the “liver and intrahepatic bile ducts” decreased, the SMR for stomach cancer increased (Table 1). However, the changes were generally small, and no different conclusions on the statistical significance of SMR were drawn at the significant level of 0.05. Again, among cancers with a specific three-digit ICD-9 code and with observed deaths, only cancers of the “liver and intrahepatic bile ducts” had a statistically significant SMR (0.49, *p* = 0.02), indicating a decreased risk. 

As a lower than expected all-cause SMR is regarded as an indicator of a healthy worker effect [9], we calculated SPMRs using data on deaths among workers for comparison (Table 2). With 52 cancer deaths, the SPMR for all cancers combined was significantly elevated (1.46, *p* = 0.01). Among cancers with a specific three-digit ICD-9 code and with observed deaths, “gallbladder and extrahepatic bile ducts” cancer had the highest SPMR (4.60, *p* = 0.14), while “liver and intrahepatic bile ducts” cancers had the lowest SPMR (1.23, *p* = 0.51). With eight deaths, stomach cancer had the only elevated SPMR (2.94) with statistical significance (*p* = 0.01), indicating a higher than expected (increased) risk. 

When person-years within the first five years of employment and the 12 cancer deaths that occurred with these periods were excluded, the adjusted SPMR for all cancer combined was 1.47 (*p* = 0.03) (Table 2). However, changes in the SPMRs of specific cancers varied. For example, the SPMRs for cancers of “lip, oral cavity, and pharynx” and “respiratory and intrathoracic organs” increased, but that for cancers of “digestive organs and peritoneum” decreased. Even among cancers of the “digestive organs and peritoneum,” whereas the SPMR for cancers of the “liver and intrahepatic bile ducts” decreased, the SPMR for stomach cancer increased (Table 2). Again, the changes were generally small, and no different conclusions on the statistical significance of SPMRs were drawn at the significant level of 0.05. With seven deaths, the stomach cancer was the only cancer with a specific three-digit ICD-9 code that had a statistically significant adjusted SPMR (3.24, *p* = 0.01), indicating an increased risk.

## 4. Discussion

In the 1980s, several studies on steel workers observed increased risks of laryngeal cancer in those who were exposed to acid mists, mainly sulfuric acid from pickling operations [5,10,11,12]. The result was later confirmed by studies on other steel workers [9,13], as well as other workers with exposure to acid mists from other manufacturing process [14]. An increased risk of lung cancer was also observed in steel workers [4,13], as well as other occupational groups with exposure to acid mists from different processes, such as soap production [14] and phosphate fertilizer production [15]. The IARC regarded the evidence for a carcinogenic effect on humans as sufficient and classified acid mists as a Class I carcinogen [2]. However, the regulatory standard of 1000 μg/m^3^ was determined on the basis of symptoms (stimulation), not the carcinogenic effect. Therefore, it is not surprising that carcinogenic effects were observed below this level [4,5], and it is important to assess the cancer risk associated with low level exposures.

The SMR for all causes combined of the study cohort was significantly smaller than 1 (*p* = 0.01), which indicated a healthy worker effect, because the overall mortality of the cohort members was lower than the general population of the same sex and age group in Taiwan [10]. Such a health worker effect is common in studies on occupational cohorts and may be introduced by various causes. In previous studies on the association between acid mists and cancer, SMRs for all causes combined ranged from 0.7 to 1.0 in most cases [3,4,14,15,16,17], indicating that a healthy worker effect generally occurred in those studies. The telecommunication company studied in this case was a government-owned enterprise, and in addition to the health examination at the beginning of their employment, employees were covered by a health insurance plan offered exclusively to government employees. In comparison with the general population in Taiwan (standard population), they had better health care, which might contribute to the healthy worker effect. Whereas the SMR for all cancer combined was significantly smaller than 1 (*p* = 0.01), the SPMR, which is much less affected by the healthy worker effect [8,18], was significantly larger than 1 (*p* = 0.01). This observation demonstrates that the health worker effect might lead to underestimation of the risk. In most previous studies on the association between acid mists and cancer, the relative risk of all cancers combined ranged from 0.7 to 1.0 [3,4,14,15,16,17], and therefore cancer risks were generally underestimated due to the healthy worker effect in most of those studies, even though some of them still observed significantly increased risks for certain cancers. Whereas the health worker effect is common in studies on occupational cohorts, not many studies took measures to control or adjust for it. The analysis in this case study demonstrated that erroneous conclusions might be reached if this effect is not taken into consideration. 

Many previous studies on acid mists did not discount the latency period of cancer. A study showed that if members with employee histories less than five years were not excluded, the excess cancer risks would not be observed [3]. Another study showed that the relative risk of laryngeal cancer generally increased after a latency period of five years was taken into account [9]. As cancer cases that occurred in the first five years of employment were not likely to be attributable to occupational exposures, the increased estimates of relative risks after adjusting for the latency period supported an association between occupation and cancer. Therefore, cancer risks associated with acid mists could have been underestimated if latency was not taken into account. After adjustment for the estimated five-year latency period, estimates of the cancer risks generally increased in our analysis, with the SMR increasing from 0.64 to 0.68 and SPMR increasing from 1.46 to 1.47. Nonetheless, the changes were small and did not affect the conclusion.

Smoking is an important risk factor for many types of cancer. We were unable to assess effects of smoking in this study directly, but a questionnaire survey in the original project found 26.8% of workers were smokers, lower than the prevalence of smoking in the general Taiwan population aged above 16 years old, which ranged from 28.21% to 33.87% from 1971 to 1996 [7]. The lower prevalence of smoking in the study cohort might also contribute to the healthy worker effect observed, as well as the observation of no increase in laryngeal or lung cancer, in our study. 

Exposure to acid mists might be affected by the distance from the workplace to the battery room, the number and type of batteries used in the building, and other factors. However, these factors were not assessed in this study, and thus we were unable to conduct a dose-response assessment. On the other hand, because the exposure to acid mists was generally low in comparison with previous studies that observed positive associations between lung or laryngeal cancers and exposure to acid mists, the results of our analysis were not in conflict with the observations in those studies. 

An increased risk of stomach cancer was observed in our study cohort. When the first five years of employment were excluded, the SPMR was 3.24 (*p* = 0.01). Using “telecommunication” or “telephone” combined with “stomach neoplasm,” “stomach cancer,” or “gastric cancer” as key words to search the literature through PubMed, we failed to find any literature on the association between working in the telecommunication industry and the occurrence of stomach cancer since 1966. Studies on the association between occupational exposure to acid mists and the occurrence of stomach cancer were quite limited. Some of the studies on associations between acid mists and cancers that we reviewed did not include stomach cancer, and others observed only one or two cases [4,14,16], which provided a limited study power. Therefore, none of them observed an increased risk of stomach cancer. In a study on battery manufacturers and steelworkers, the 2678 workers who were “definitely exposed to acid mists” had a relative risk of 1.2 for stomach cancer, in comparison with the 1356 who were “never exposed to acid mists” [3]. A case-control study on cancer of the gastric cardia conducted through reviewing death certificates from 24 states in the U.S. from 1984 to 1992 found a consistent pattern of risk increase by intensity (“low,” “medium,” and “high”; *p* < 0.01 for test for trend) and probability (“low,” “medium,” and “high”; *p* < 0.05 for test for trend) of exposure to sulfuric acid mists, with a two-fold excess associated with a high probability of high-intensity exposure [19]. When all exposed workers were put together, an increased relative risk of 1.2 (*p* ≈ 0.05) was also observed. In a follow-up of that study, which studied gastric cancers as a whole with four more years of data (from 1984 to 1996), an increasing trend of risk with probability of exposure was observed in workers who had high-intensity of exposure only (*p* < 0.01), and an increasing trend of risk with intensity of exposure was observed in all exposed workers combined (*p* < 0.01) and in those who had a medium probability of exposure (*p* < 0.05) [20]. Workers with a high probability and high intensity of exposure had a relative risk of 1.29 (*p* > 0.05). The lower consistency in the trends of risk associated with exposure observed in the follow-up study might indicate that the association between acid mists and stomach cancer was more specific to cancer of the gastric cardia. However, the death registry we used did not provide enough information on diagnoses for us to test this hypothesis. A study in the U.S. observed increased risks of stomach cancer in 4518 workers of lead battery plants (SMR = 1.68, *p* < 0.01) and 2300 workers of lead smelters (SMR = 1.46, *p* > 0.05) [21]. A follow-up of this study with a longer duration of observation confirmed the observations, and the SMR for stomach cancer was 1.528 (*p* < 0.01) in lead battery plant workers and 1.334 (*p* > 0.05) in lead smelter workers [22]. No dose-response relationship between lead exposure and mortality from stomach cancer was observed. Whereas exposure levels to acid mists were not reported for that cohort, judging from the fact that lead battery manufacturing is an industry with high acid mist exposure, while lead smelting is not [22], the results of these two studies support the association between occupational acid mist exposure and stomach cancer. Although some evidence showed that gastroesophageal reflux was an important risk factor of stomach cancer [23], it is unclear whether inhalation and subsequent ingestion of acid mists can cause similar effects. Since low pH can increase instability in chromosomes and DNA, it has been proposed that acid mists cause cancers through genotoxic effects [24]. 

In addition to tobacco smoking, Helicobacter pylori, X-radiation, and gamma-radiation were also recognized as Class I carcinogens of the stomach, as well as the rubber production industry. In Taiwan, ionizing radiation in the work place is under strict surveillance by the government, and no excess exposure to ionizing radiation had been reported in the buildings we studied. Regarding Helicobacter pylori, there was no evidence in the literature showing that working in the telecommunication industry or being in buildings with battery rooms is associated with a higher prevalence of Helicobacter pylori infection. Therefore, there was no evidence supporting that the higher risk of stomach cancer observed in our analysis was attributable to these potential confounders. On the other hand, we do not have certainty about the cause of the observed excess mortality due to stomach cancer in this working population, and the finding must be confirmed by further studies. Such studies may include internal comparisons within the study population (including dose-response assessments), which is also an approach commonly applied to minimize the impact of healthy worker effects.

## 5. Conclusions

In epidemiological studies, it is not uncommon that the study population is relatively healthier than the comparison population, especially when the study population is an occupational population and the comparison population is a sample of the general population. Using a study on the mortality of telecommunication workers as an example, we have demonstrated that the risk associated with the exposure would be underestimated due to the healthy worker effect and that using SPMR is more appropriate than SMR in terms of risk assessment. In this case study, working in buildings with battery rooms was found to be associated with increased mortalities due to all cancer combined and stomach cancer. Whereas exposure to acid mist might have contributed to the increased risks, no personal exposure data were obtained, and no control of other occupational risk factors was made. Furthermore, because the study population was relatively healthy, we did not identify a large number of cancer deaths, which made comparisons among subgroups within the study cohort not feasible. Therefore, further studies should be conducted to evaluate whether the increased risks are related to low-level exposure to acid mists or other factors in the work environment.

## Figures and Tables

**Table 1:** Standardized mortality ratios (SMRs) of various causes of death from 1985 to 1995.

Causes of Death	Crude Data			Adjusted for 5 Years Latency		
	Cases	SMR	*p*	Cases	SMR	*p*
All causes	155	0.42	<0.01	122	0.46	<0.01
All cancers	52	0.64	<0.01	42	0.68	<0.01
Lip, oral cavity, and pharynx	5	0.39	0.03	5	0.50	0.13
Nasopharynx	5	0.79	0.80	5	1.05	>0.99
Digestive organs and peritoneum	31	0.71	0.06	23	0.69	0.07
Esophagus	1	0.37	0.51	0		
Stomach	8	1.42	0.41	7	1.64	0.28
Colon	3	0.91	>0.99	2	0.80	>0.99
Rectum and rectosigmoid junction	2	0.97	>0.99	2	1.24	0.96
Liver and intrahepatic bile ducts	14	0.52	0.01	10	0.49	0.02
Gallbladder and extrahepatic bile ducts	2	2.11	0.49	1	1.33	>0.99
Pancreas	1	0.61	>0.99	1	0.77	>0.99
Respiratory and intrathoracic organs	7	0.60	0.21	7	0.76	0.59
Trachea, bronchus, and lung	7	0.67	0.37	7	0.84	0.81
Cervix uteri	1	3.34	0.52	1	5.77	0.32
Kidney and other and unspecified urinary organs	1	1.27	>0.99	1	1.61	0.92
Thyroid gland	1	7.20	0.26	1	9.49	0.20
Other endocrine glands and related structures	1	7.67	0.24	1	9.92	0.19
Other and ill-defined sites	1	8.47	0.22	1	9.77	0.19
Multiple myeloma and immunoproliferative neoplasms	1	3.94	0.45	0		
Myeloid leukemia	2	1.55	0.74	1	1.16	>0.99
Leukemia of unspecific cell type	1	1.03	>0.99	1	1.44	>0.99
Accident	33	0.28	<0.01	27	0.36	<0.01
Cerebrovascular diseases	16	0.55	0.01	13	0.58	0.04
Cardiovascular disease	16	0.65	0.08	12	0.64	0.13
Diabetes mellitus	9	1.02	>0.99	8	1.10	0.87

**Table 2:** Standardized proportional mortality ratios (SPMRs) for cancers from 1985 to 1994.

Causes of Death	Crude Data			Adjust for 5 Years of Latency		
	Cases	SPMR	*p*	Cases	SPMR	*p*
All cancers	52	1.46	0.01	42	1.47	0.03
Lip, oral cavity, and pharynx	5	0.95	>0.99	5	1.18	0.83
Nasopharynx	5	1.99	0.22	5	2.49	0.11
Digestive organs and peritoneum	31	1.61	0.02	23	1.48	0.09
Esophagus	1	0.78	>0.99	0		
Stomach	8	2.94	0.01	7	3.24	0.01
Colon	3	2.01	0.38	2	1.66	0.68
Rectum and rectosigmoid junction	2	2.13	0.48	2	2.59	0.36
Liver and intrahepatic bile ducts	14	1.23	0.51	10	1.09	0.87
Gallbladder and extrahepatic bile ducts	2	4.60	0.14	1	2.79	0.60
Pancreas	1	1.31	>0.99	1	0.57	0.94
Respiratory and intrathoracic organs	7	1.21	0.73	7	1.46	0.41
Trachea, bronchus, and lung	7	1.33	0.56	7	1.60	0.30
Cervix uteri	1	10.79	0.18	1	10.79	0.18
Kidney and other and unspecified urinary organs	1	2.66	0.63	1	3.26	0.53
Thyroid gland	1	16.06	0.12	1	19.38	0.10
Other endocrine glands and related structures	1	17.93	0.11	1	21.87	0.09
Other and ill-defined sites	1	20.21	0.10	1	22.79	0.09
Multiple myeloma and immunoproliferative neoplasms	1	8.70	0.22	0		
Myeloid leukemia	2	4.17	0.17	1	2.67	0.62
Leukemia of unspecific cell type	1	2.74	0.61	1	3.33	0.52
Accident	33	0.77	0.14	27	0.85	0.45
Cerebrovascular diseases	16	1.15	0.63	13	1.18	0.63
Cardiovascular disease	16	1.40	0.24	12	1.31	0.42
Diabetes mellitus	9	2.11	0.06	8	2.24	0.06

## Data Availability

The data presented in this study are available upon reasonable requests.
